# Protein Kinase D1 (PRKD1) as a Diagnostic, Prognostic, and Immunomodulatory Biomarker in Human Cancers

**DOI:** 10.7759/cureus.90765

**Published:** 2025-08-22

**Authors:** Ruba Abdelrazig, Khansa Ali, M. Almontaser H Abubaker, Nabila Issa Youssouf, Salah A Alshikh, Samah Mustafa Mohammedosman, Mohamed Alfaki

**Affiliations:** 1 Department of Pharmacy, University of Khartoum, Khartoum, SDN; 2 Department of Microbiology, University of Gezira, Madani, SDN; 3 Department of Medical Laboratory Sciences, University of Kordofan, Al-Ubayyid, SDN; 4 Faculty of Medicine and Surgery, International University of Africa, Khartoum, SDN; 5 Department of Medicine and Surgery, Hayatt University College, Khartoum, SDN; 6 Department of Medicine and Health Sciences, Red Sea University, Port Sudan, SDN; 7 Department of Computer Science, Al Neelain University, Khartoum, SDN

**Keywords:** bioinformatics, biomarkers, cancer genetics, diagnostic biomarker, genetics, pan cancer analysis, prkd1, prognostic biomarker

## Abstract

Background and aim: Protein kinase D1 (PRKD1), a serine/threonine kinase, regulates cellular processes such as proliferation, apoptosis, migration, and immune responses. Depending on the tumor context, PRKD1 exhibits either oncogenic or tumor-suppressive functions. This study aimed to delineate the role of PRKD1 in cancer progression and assess its diagnostic and prognostic potential across multiple cancer types.

Materials and methods: We analyzed PRKD1 expression using Tumor Immune Estimation Resource (TIMER), Gene Expression Profiling Interactive Analysis (GEPIA), and University of ALabama at Birmingham CANcer data analysis Portal (UALCAN) databases, and assessed its prognostic significance via the Kaplan-Meier plotter. Mutation profiles were examined using cBioPortal, while gene-gene and protein-protein interactions were evaluated through GeneMANIA and STRING, respectively. Pathway enrichment was performed using Enrichr. Findings were validated using three GEO datasets: GSE24152, GSE15641, and GSE110224.

Results: PRKD1 expression was significantly downregulated in bladder urothelial carcinoma (BLCA), kidney chromophobe (KICH), and rectum adenocarcinoma (READ) (all p < 0.001). Expression levels varied significantly with clinical parameters, including age, gender, race, and tumor stage. Immune infiltration analysis revealed significant associations between PRKD1 expression and immune cell subsets, namely, B cells, CD4⁺ T cells, macrophages, neutrophils, and dendritic cells in thyroid carcinoma (THCA), stomach adenocarcinoma (STAD), liver hepatocellular carcinoma (LIHC), and kidney renal clear cell carcinoma (KIRC). A notable inverse correlation was observed between PRKD1 and CD8⁺ T cell levels in THCA (p > 0.05). Survival analysis demonstrated that low PRKD1 expression correlated with improved prognosis in STAD, THCA, and LIHC, whereas high expression was favorable in KIRC (p = 0.001). Promoter methylation of PRKD1 was significantly increased in KICH and READ and decreased in BLCA (all p < 0.001), suggesting epigenetic regulation underlies its differential expression.

Conclusion: PRKD1 serves as a potential diagnostic biomarker in BLCA, KICH, and READ, and as a prognostic indicator in STAD, THCA, LIHC, and KIRC. Its expression is modulated by epigenetic mechanisms and correlates with immune cell infiltration, underscoring its relevance in tumor immunobiology and potential as a therapeutic target.

## Introduction

Cancer remains one of the most significant global health challenges, accounting for nearly 10 million deaths in 2020, which translates to approximately one in six deaths worldwide [1]. Despite notable advances in cancer treatment and research, the precise molecular mechanisms underlying tumor development and resistance to therapy remain to be explored. Among the many factors involved, protein kinase D1 (PRKD1) has garnered attention due to its complex role in tumor biology. Interestingly, PRKD1 exhibits both tumor-suppressive and oncogenic properties, depending on the specific cellular and molecular context [2]. As a serine/threonine kinase, PRKD1 plays a crucial role in regulating cell proliferation, apoptosis, differentiation, migration, and immune system interactions [3]. However, its function in cancer is far from being straightforward. Research suggests that PRKD1 can either inhibit or promote tumor growth, with outcomes influenced by genetic factors, signaling networks, and the surrounding tumor microenvironment [4,5]. This dual nature makes PRKD1 an intriguing but challenging target for cancer prognosis and therapy. Although some studies have suggested its potential as a biomarker and therapeutic target, the extent of its involvement across different malignancies remains unclear. This study aims to comprehensively assess the diagnostic, prognostic, and immunomodulatory significance of PRKD1 across multiple cancer types. Using bioinformatics analyses of publicly available databases (Gene Expression Profiling Interactive Analysis (GEPIA), University of ALabama at Birmingham CANcer data analysis Portal (UALCAN), and Tumor Immune Estimation Resource (TIMER)), we examined gene expression patterns, immune cell infiltration, survival outcomes, and enrichment analysis. Through these approaches, we aimed to elucidate the contribution of PRKD1 to tumor progression and clinical outcomes, thereby highlighting its potential as a biomarker and therapeutic target in human cancers.

## Materials and methods

Gene expression analysis

PRKD1 gene expression across various cancer types was investigated using the Tumor Immune Estimation Resource (TIMER) (https://cistrome.shinyapps.io/timer/). It is a comprehensive resource for systematic analysis of immune infiltrates across diverse cancer types [6]. Furthermore, we used the Gene Expression Profiling Interactive Analysis (GEPIA) (http://gepia.cancerpku.cn/index.html/). It is an interactive web server for analyzing RNA sequencing expression data from The Cancer Genome Atlas (TCGA) and the Genotype-Tissue Expression (GTEx) projects [7].

Additional analysis was performed to assess PRKD1 gene expression in various cancer types using the University of ALabama at Birmingham CANcer data analysis Portal (UALCAN) database (https://ualcan.path.uab.edu/index.html). It is a comprehensive, user-friendly, and interactive web resource for analyzing cancer OMICS data. It provides in silico validation of potential genes of interest, graphs, and plots depicting expression profiles and patient survival information, as well as pan-cancer gene expression analysis [8].

Immune cell infiltration analysis

The Tumor Immune Estimation Resource (TIMER) is an extensive tool for systematically evaluating immune cell infiltrates in various cancer types [6]. In this research, we employed the TIMER database (https://cistrome.shinyapps.io/timer/) to explore the relationship between immune cell infiltrates and PRKD1 gene expression.

Prognostic significance of PRKD1 in cancers

To investigate the prognostic significance of PRKD1 expression across various cancer types, we used the Kaplan-Meier plotter (https://kmplot.com/). It is an online tool that investigates the relationship between gene expression and patient survival in various cancer types [9]. Moreover, the GEPIA database (http://gepia.cancerpku.cn/index.html/) [7] was used. To investigate the impact of PRKD1 expression on patient survival, the UALCAN database (https://ualcan.path.uab.edu/index.html) was used [8].

Genetic alteration analysis

cBioPortal for Cancer Genomics is an open-access online resource that provides a comprehensive database of cancer genomic data and tools for its exploration [10]. In this study, we used cBioPortal (https://www.cbioportal.org/) to investigate PRKD1 mutations across diverse cancer types using clinical reports.

Gene-gene interaction

GeneMANIA is an online tool that provides biological network integration for gene prioritization and prediction of gene function [11]. We utilize GeneMANIA (https://www.genemania.org/) to detect the type of interaction between PRKD1 and positively correlated genes.

Protein-protein interaction

STRING is a database of known and predicted protein-protein interactions [12]. We used STRING (https://string-db.org/) to detect protein-protein interactions for proteins that result from the expression of PRKD1 and positively correlated genes.

Enrichment analysis

Enricher is a comprehensive and interactive platform for enrichment analysis that contains updated gene set libraries, a new method for ranking enriched terms, and diverse interactive visualizations of results powered by the D3 JavaScript library [13]. Using Enricher (https://maayanlab.cloud/Enrichr/), we performed enrichment analysis to investigate the biological processes and pathways associated with PRKD1 and positively correlated genes.

Validation

To confirm our results, we use the database resources of the National Center for Biotechnology Information (NCBI) (https://doi.org/10.1093/nar/gkab1112) [14]. We compared two or more groups of samples to identify genes that are differentially expressed across experimental conditions using the GEO2R tool (https://www.ncbi.nlm.nih.gov/geo/geo2r). It is an online tool that applies statistical methods to compare sample groups and identify significantly differentially expressed genes (DEGs) [15]. R was then used to create volcano plots to visualize the differential expression profiles.

## Results

Gene expression analysis data

**Figure 1:**
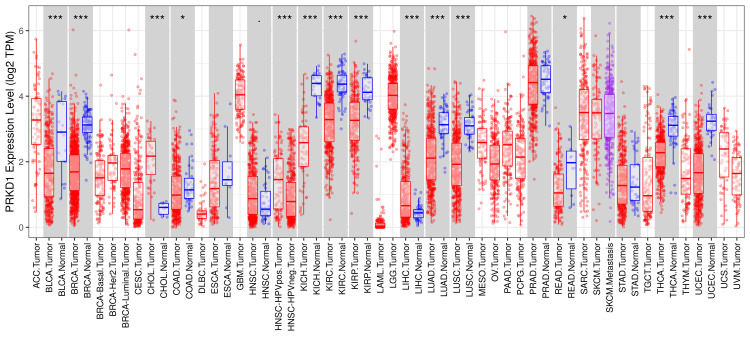
PRKD1 expression levels in tumor and normal samples across various cancer types using TIMER 1 This figure demonstrates the expression levels of PRKD1 in different types of cancers. PRKD1 levels were significantly different from normal tissue in 14 cancer types: BLCA, BRCA, CHOL, COAD, HNSA-HPVpos and HNSA-HPVneg, KICH, KIRC, KIRP, LICH, LUAD, LUSC, READ, THCA, and UCEC. *p < 0.05, **p < 0.01, ***p < 0.001 Red: tumor samples, blue: normal samples ACC: adrenocortical carcinoma,  BLCA: bladder urothelial carcinoma, BRCA: breast invasive carcinoma, Her2: human epidermal growth factor 2, CESC: cervical squamous cell carcinoma and endocervical adenocarcinoma, CHOL: cholangiocarcinoma, COAD: colon adenocarcinoma, DLBC: lymphoid neoplasm diffuse large B-cell lymphoma, ESCA: esophageal carcinoma, GBM: glioblastoma multiforme, HNSC: head and neck squamous cell carcinoma, HPVpos: human papillomavirus positive, HPVneg: human papillomavirus negative, KICH: kidney chromophobe, KIRC: kidney renal clear cell carcinoma, KIRP: kidney renal papillary cell carcinoma, LAML: acute myeloid leukemia, LGG: brain lower grade glioma, LIHC: liver hepatocellular carcinoma, LUAD: lung adenocarcinoma, LUSC: lung squamous cell carcinoma, MESO: mesothelioma, OV: ovarian serous cystadenocarcinoma, PAAD: pancreatic adenocarcinoma, PCPG: pheochromocytoma and paraganglioma, PRAD: prostate adenocarcinoma, PRKD1: protein kinase D1, READ: rectum adenocarcinoma, SARC: sarcoma, SKCM: skin cutaneous melanoma, STAD: stomach adenocarcinoma, TGCT: testicular germ cell tumors, THCA: thyroid carcinoma, THYM: thymoma, TIMER 1: Tumor Immune Estimation Resource, UCEC: uterine corpus endometrial carcinoma, UCS: uterine carcinosarcoma, UVM: uveal melanoma

PRKD1 expression levels were evaluated utilizing TIMER, GEPIA, and UALCAN databases. Initial screening using TIMER 1 allowed for a thorough comparison of gene expression patterns in diverse cancers. The results indicated that PRKD1 levels deviated significantly from normal tissue in 14 cancer types. Among these, 12 exhibited pronounced downregulation of PRKD1: bladder urothelial carcinoma (BLCA), breast invasive carcinoma (BRCA), colon adenocarcinoma (COAD), head and neck squamous cell carcinoma (HNSC), kidney chromophobe (KICH), kidney renal papillary cell carcinoma (KIRP), kidney renal clear cell carcinoma (KIRC), lung adenocarcinoma (LUAD), lung squamous cell carcinoma (LUSC), rectum adenocarcinoma (READ), thyroid carcinoma (THCA), and uterine corpus endometrial carcinoma (UCEC). In contrast, only two cancers, cholangiocarcinoma (CHOL) and liver hepatocellular carcinoma (LICH), showed significant PRKD1 upregulation (Figure 1).

**Figure 2:**
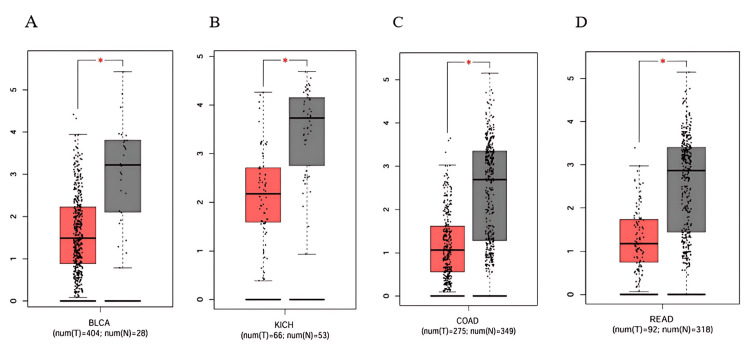
PRKD1 expression levels in tumor and normal samples across various cancer types using GEPIA 1 (A) PRKD1 levels in BLCA. (B) PRKD1 levels in COAD. (C) PRKD1 levels in KICH. (D) PRKD1 levels in READ. *p < 0.05 Red: tumor samples, gray: normal samples BLCA: bladder urothelial carcinoma, COAD: colon adenocarcinoma, GEPIA: Gene Expression Profiling Interactive Analysis, KICH: kidney chromophobe, PRKD1: protein kinase D1, READ: rectum adenocarcinoma

Subsequently, we analyzed PRKD1 expression in 14 different cancers using the GEPIA 1 database, sourced from TIMER 1. We observed that PRKD1 expression level was consistently and significantly reduced in BLCA, COAD, KICH, and READ tissues compared to healthy tissues (Figure 2). However, no notable differences in PRKD1 expression were found between normal and tumor tissues in BRCA, CHOL, HNSC, KIRC, KIRP, LIHC, LUSC, LUAD, THCA, and UCEC (Appendices).

**Figure 3:**
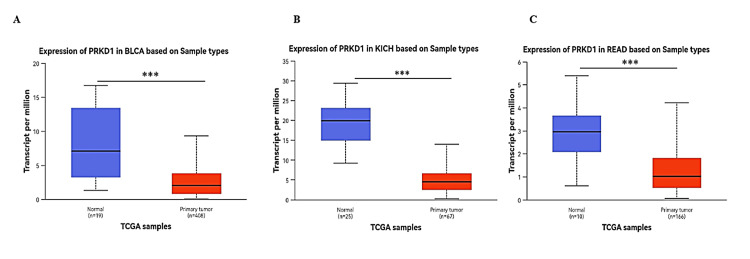
PRKD1 expression levels in tumor and normal samples across various cancer types using UALCAN (A) BLCA. (B) KICH. (C) READ. *p < 0.05, **p < 0.01, ****p < 0.0001 Red: tumor samples, blue: normal samples BLCA: urothelial bladder cancer, KICH: chromophobe renal carcinoma, N: normal, num: number, PRKD1: protein kinase D1, READ: colorectal adenocarcinoma, T: tumor, TCGA: The Cancer Genome Atlas, UALCAN: University of ALabama at Birmingham CANcer data analysis Portal

Lastly, we employed the UACLAN database to examine the four cancer types consistently downregulated and shared between TIMER 1 and GEPIA 1 databases. PRKD1 expression was markedly reduced in BLCA, KICH, and READ tissues compared to healthy tissues (all p > 0.001) (Figure 3). Conversely, PRKD1 expression in COAD tissues showed no significant difference from normal tissues (Appendices). We conducted further analyses on these three cancer types: BLCA, KICH, and READ.

PRKD1 was found to be significantly downregulated in three cancer types (BLCA, READ, and KICH) based on TIMER, GEPIA, and UALCAN databases. These cancers were subsequently prioritized for further investigations, including clinical parameter correlations, methylation profiling, gene-gene interaction, protein-protein interaction, and enrichment analyses.

Analysis of PRKD1 expression across clinical variables

**Figure 4:**
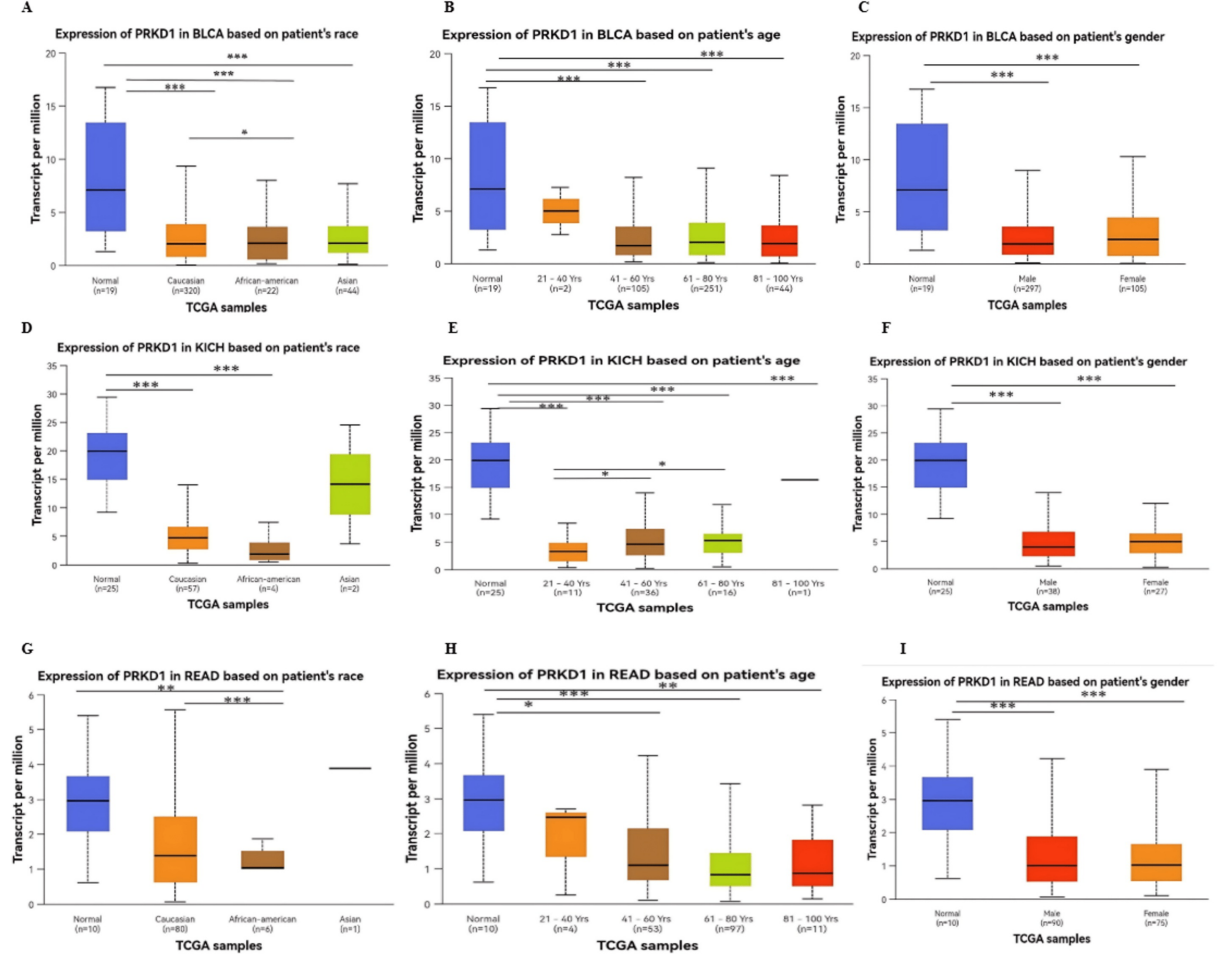
PRKD1 expression levels in cancer patients according to specific clinical variables using UALCAN (A-C) Expression of PRKD1 in BLCA patients according to (A) race, (B) age, and (C) gender. (D-F) Expression of PRKD1 in KICH patients according to (D) race, (E) age, and (F) gender. (G-I) Expression of PRKD1 in READ patients according to (G) race, (H) age, and (I) gender. *p < 0.05, ***p < 0.001, ****p < 0.0001 BLCA: bladder urothelial carcinoma, KICH: kidney chromophobe, PRKD1: protein kinase D1, READ: rectum adenocarcinoma, TCGA: The Cancer Genome Atlas, UALCAN: University of ALabama at Birmingham CANcer data analysis Portal

The expression levels of PRKD1 were assessed in relation to various clinical factors using data from TIMER 1, GEPIA 1, and UALCAN databases. In three cancer types (BLCA, KICH, and READ), PRKD1 expression was consistently and significantly low compared to healthy tissues. The study explored PRKD1 expression in these cancers by categorizing patients based on age, sex, race, and cancer stage. Age groups were divided into young adults (21-40 years), middle-aged adults (41-60 years), older adults (61-80 years), and elderly individuals (81-100 years). Sex was categorized as male or female, while race included Caucasian, African American, and Asian groups. Results indicated that PRKD1 expression was significantly different across distinct age, sex, and racial categories in BLCA, KICH, and READ (Figure 4). Notably, in BLCA, Caucasian patients showed significantly higher PRKD1 expression than African American patients (p < 0.05) (Figure 4A). Additionally, PRKD1 expression was significantly lower in middle-aged adults (41-60 years, p < 0.01), older adults (61-80 years, p < 0.01), and elderly individuals (81-100 years, p < 0.05) compared to healthy controls, but young adults (21-40 years) showed no significant difference (Figure 4B). PRKD1 expression did not differ significantly between male and female patients in BLCA (Figure 4C).

In KICH, there was no significant difference in PRKD1 expression between race groups (Figure 4D). Furthermore, PRKD1 expression was significantly reduced in young adults (21-40 years), middle-aged adults (41-60 years), and older adults (61-80 years) (all p < 0.0001). However, older adults (61-80 years) did not differ significantly from healthy controls, while middle-aged adults (41-60 years, p < 0.05) and older adults (61-80 years, p < 0.05) showed significant differences compared to young adults (Figure 4E). PRKD1 expression did not differ significantly between male and female patients (Figure 4F).

Similarly, in READ, Caucasian patients exhibited significantly higher PRKD1 expression than African American patients (p < 0.001, Figure 4G). Moreover, PRKD1 expression was significantly lower in middle-aged adults (41-60 years, p < 0.05), older adults (61-80 years, p < 0.001), and elderly individuals (81-100 years, p < 0.01) compared to healthy controls, but young adults (21-40 years) showed no significant difference (Figure 4H). No significant difference in PRKD1 expression was observed between male and female groups (Figure 4I).

**Figure 5:**
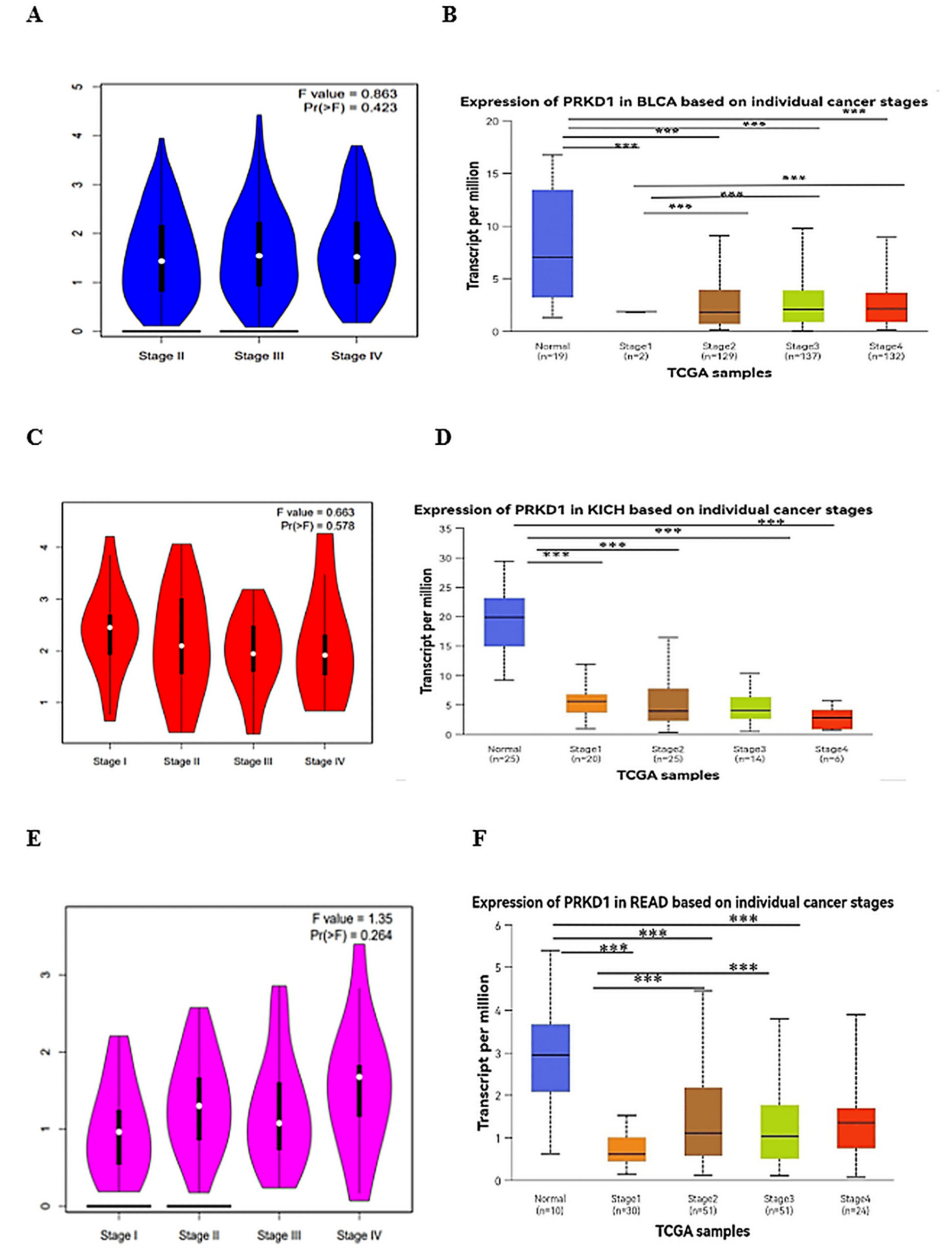
PRKD1 expression analysis in cancer patients according to cancer stage using GEPIA 1 and UALACAN (A and B) Expression of PRKD1 in BLCA patients according to cancer stage. (C and D) Expression of PRKD1 in KICH patients according to cancer stage. (E and F) Expression of PRKD1 in READ patients according to cancer stage. *p < 0.05, **p < 0.01, ***p < 0.001, ****p < 0.0001 BLCA: bladder urothelial carcinoma, KICH: kidney chromophobe, PRKD1: protein kinase D1, READ: rectum adenocarcinoma, TCGA: The Cancer Genome Atlas, UALCAN

Our study examined PRKD1 gene expression across various cancer stages using data from the GEPIA 1 database, which relies on TCGA clinical data [7], and the UALCAN database, which uses American Joint Committee on Cancer (AJCC) staging [8]. GEPIA 1 data analysis identified significant variations in PRKD1 expression across clinical stages for BLCA and KICH (all p < 0.05, Figure 5A, 5C), but no notable differences for READ (Figure 5E). UALCAN database analysis revealed a significant reduction in PRKD1 expression in all BLCA and KICH stages compared to normal tissue, with BLCA stage I differing significantly from stages II, III, and IV (all p < 0.0001, Figure 5B), while KICH showed no significant differences between stages (Figure 5D). For READ, all stages except stage IV exhibited significantly lower PRKD1 expression than normal tissue, with stage I differing significantly from stage II (p < 0.05) and stage III (p > 0.01, Figure 5F).

PRKD1 expression correlation with immune cell recruitment and infiltration in four cancer types

**Figure 6:**
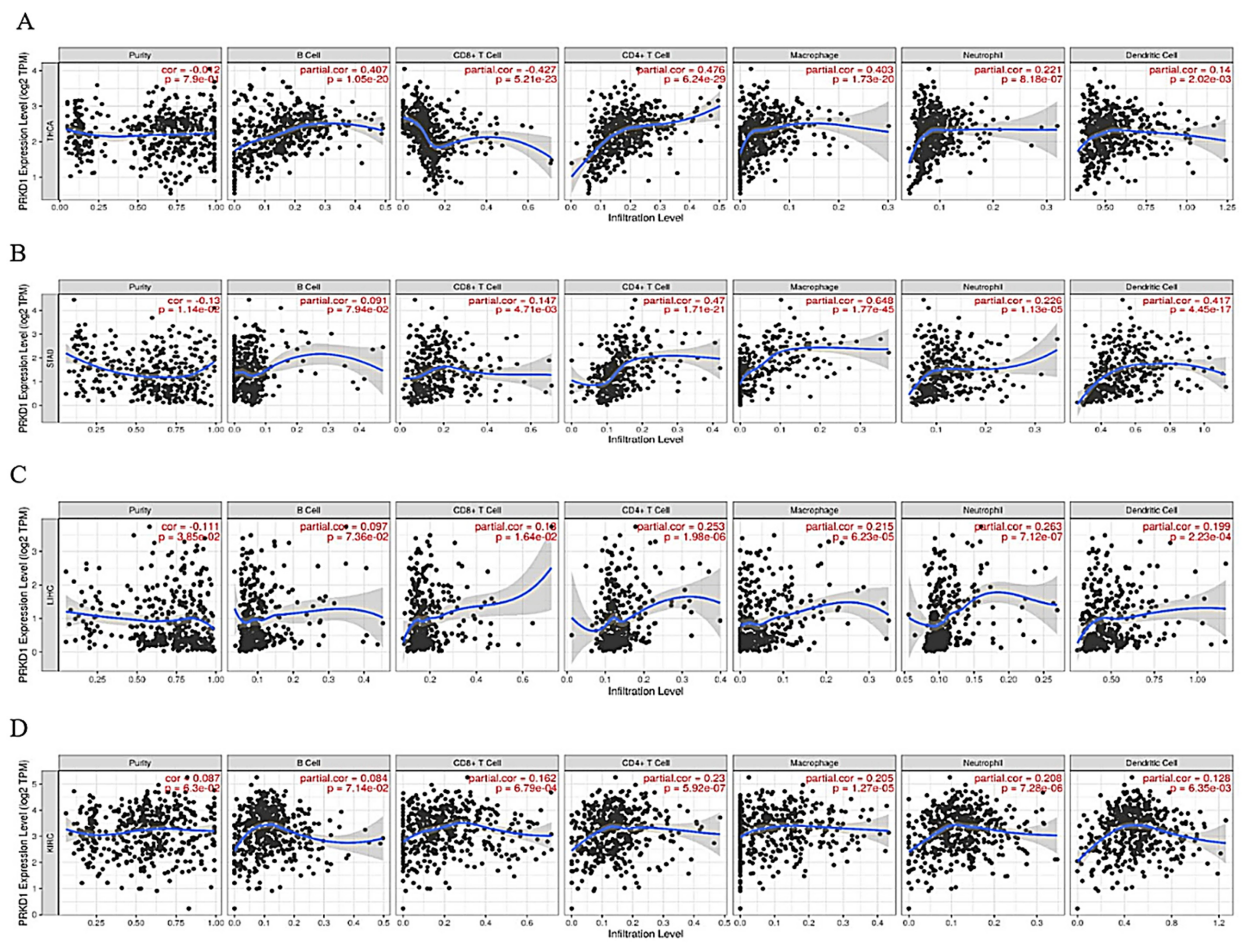
Correlation of PRKD1 gene expression with immune cell recruitment across various cancer types using the TIMER 1 The scatterplots of correlations between PRKD1 expression and immune cell infiltration in (A) THCA, (B) STAD, (C) LIHC, and (D) KIRC. KIRC: kidney renal clear cell carcinoma, LIHC: liver hepatocellular carcinoma, PRKD1: protein kinase D1, STAD: stomach adenocarcinoma, THCA: thyroid carcinoma

We investigated the relationship between PRKD1 gene expression and the infiltration levels of immune cells, involving B cells, CD8+ T cells, CD4+ T cells, macrophages, neutrophils, and dendritic cells, using TIMER 1. In THCA patients, PRKD1 expression showed a significant negative correlation with CD8+ T cell infiltration (Cor = -0.427, p = 5.21e-23), but positive correlations with B cells (Cor = 0.407, p = 1.25e-20), CD4+ T cells (Cor = 0.476, p = 6.24e-29), macrophages (Cor = 0.403, p = 1.7e-20), neutrophils (Cor = 0.221, p = 8.18e-07), and dendritic cells (Cor = 0.14, p = 2.02e-03) (Figure 6A).

In STAD, PRKD1 expression was positively correlated with B cells (Cor = 0.091, p = 7.94e-02), CD8+ T cells (Cor = 0.147, p = 4.71e-03), CD4+ T cells (Cor = 0.47, p = 1.71e-21), macrophages (Cor = 0.648, p = 1.77e-45), neutrophils (Cor = 0.226, p = 1.13e-05), and dendritic cells (Cor = 0.417, p = 4.45e-17); however, the correlation with B cells was not statistically significant (Figure 6B). Similarly, in LICH patients, PRKD1 expression was positively correlated with B cells (Cor = 0.097, p = 7.36e-02), CD8+ T cells (Cor = 0.18, p = 1.64e-02), CD4+ T cells (Cor = 0.253, p = 1.98e-06), macrophages (Cor = 0.215, p = 6.23e-05), neutrophils (Cor = 0.263, p = 7.12e-07), and dendritic cells (Cor = 0.199, p = 2.23e-04), with no significant correlation for B cells (Figure 6C).

In KIRC patients, PRKD1 expression was positively correlated with B cells (Cor = 0.087, p = 7.14e-02), CD8+ T cells (Cor = 0.162, p = 6.79e-04), CD4+ T cells (Cor = 0.23, p = 5.92e-07), macrophages (Cor = 0.205, p = 1.27e-05), neutrophils (Cor = 0.208, p = 7.28e-6), and dendritic cells (Cor = 0.128, p = 6.35e-03) (Figure 6D). Overall, PRKD1 expression was positively correlated with immune cell infiltration across the four cancers, except for a negative correlation with CD8+ T cells in THCA and non-significant correlations with B cells in STAD and LICH.

PRKD1 as a potential prognostic biomarker

**Figure 7:**
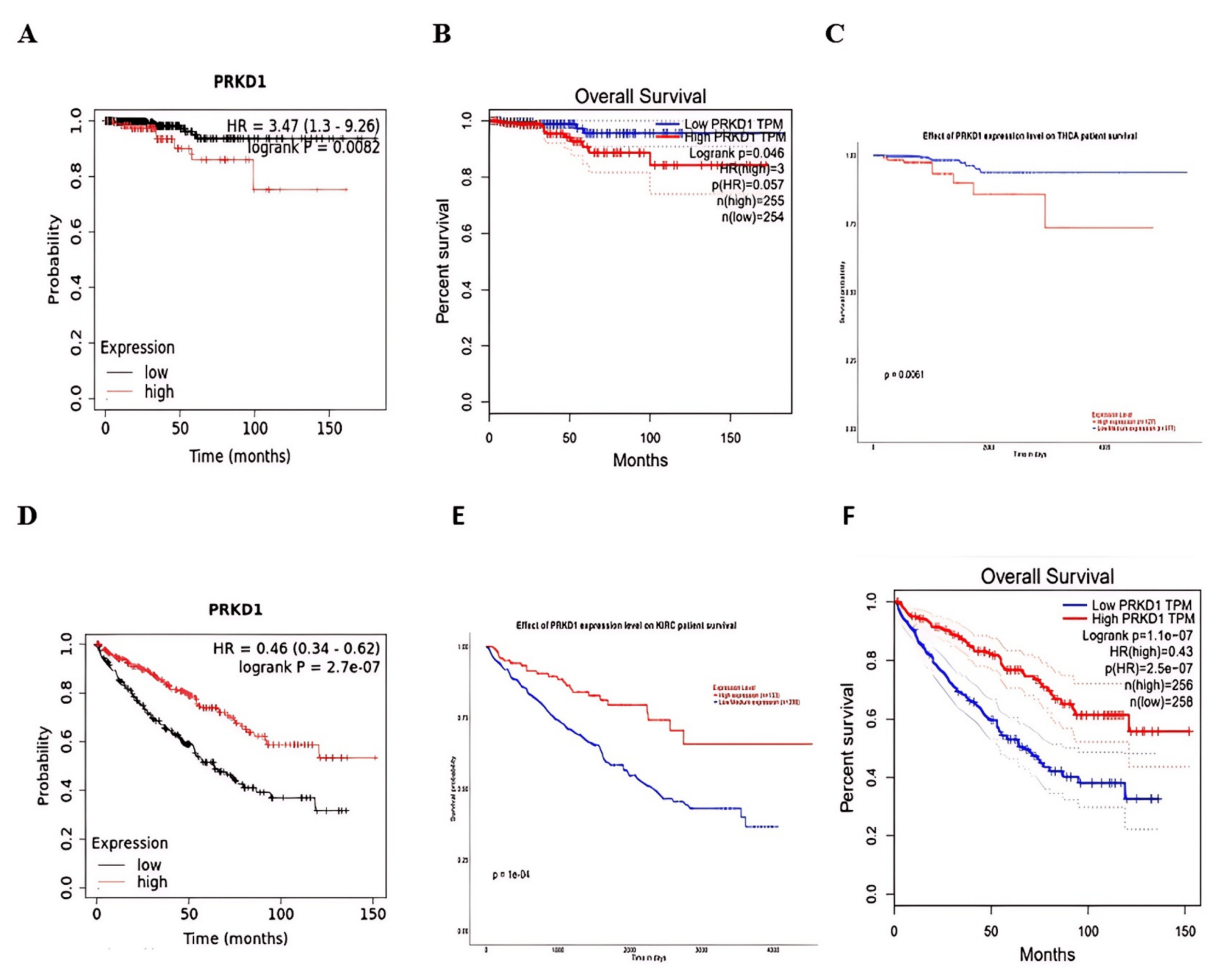
Association between PRKD1 expression levels and overall survival in THCA and KIRC patients Survival analysis indicated a significant prognostic role of THCA and KIRC. (A-C) OS of PRKD1 in THCA according to (A) Kaplan-Meier, (B) GEPIA 1, and (C) UALCAN. (D and E) OS of PRKD1 in KIRC according to (D) Kaplan-Meier, (E) GEPIA 1, and (F) UALCAN. Red line: high gene expression, blue and black line: low gene expression GEPIA: Gene Expression Profiling Interactive Analysis, KIRC: kidney renal clear cell carcinoma, OS: overall survival, PRKD1: protein kinase D1, THCA: thyroid carcinoma, UALCAN: University of ALabama at Birmingham CANcer data analysis Portal

Survival analysis was conducted using data from Kaplan-Meier plotter, GEPIA, and UALCAN databases to evaluate the relationship between PRKD1 expression and overall survival (OS) in patients. The findings indicated that reduced PRKD1 expression was strongly linked to a favorable prognosis in THCA patients (Kaplan-Meier plotter: HR = 3.47 (95% CI: 1.3-9.2), p=0.0082, Figure 7A; GEPIA: p = 0.04, Figure 7B; UALCAN: p = 0.006, Figure 7C), while elevated PRKD1 expression was significantly associated with a favorable prognosis in KIRC patients (Kaplan-Meier plotter: HR = 0.46 (95% CI: 0.34-0.62), p = 2.7e-07, Figure 7D; GEPIA: p = 1.1e-07, Figure 7E; UALCAN: p = 1.1e-04, Figure 7F).

**Figure 8:**
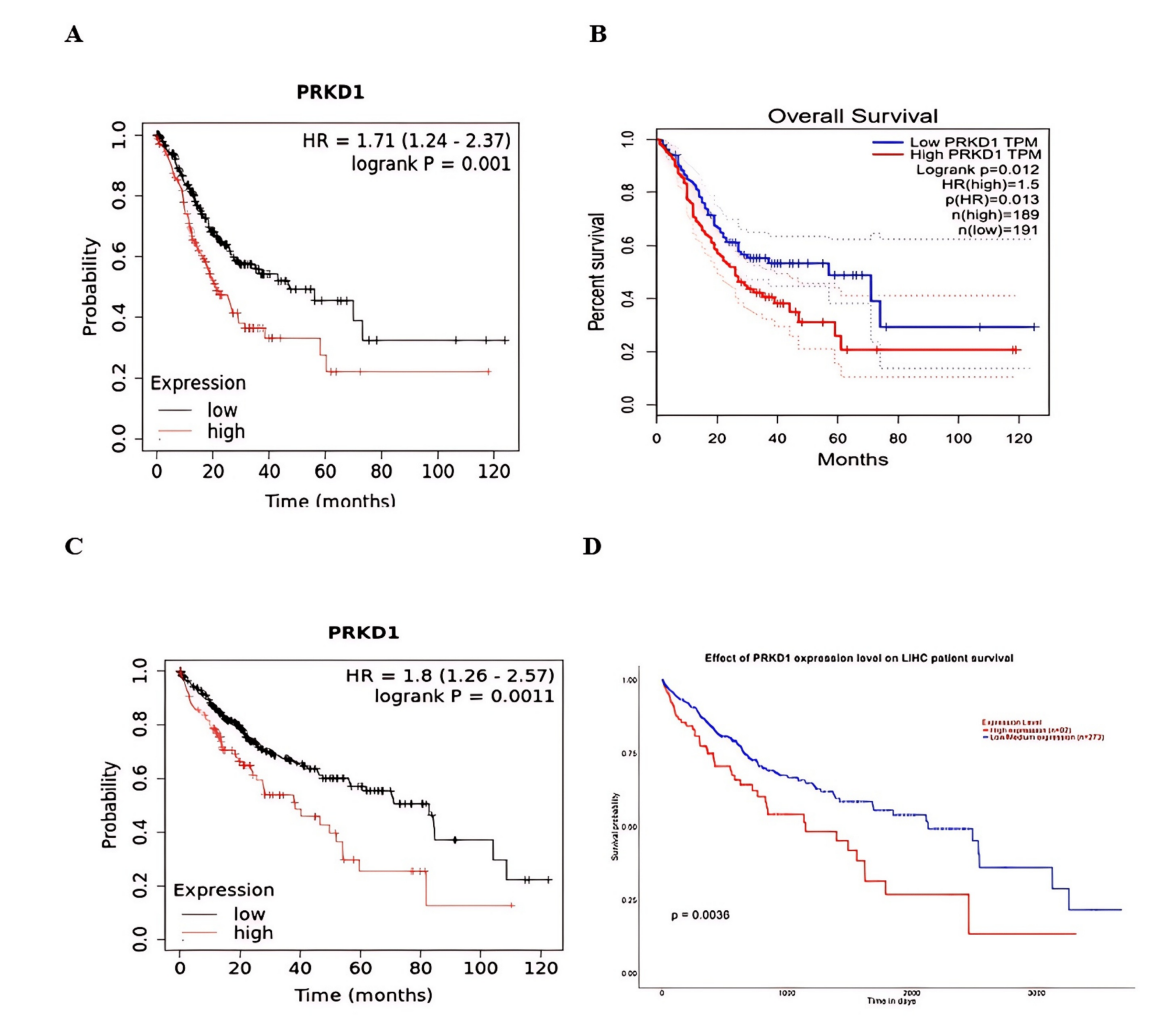
Association between PRKD1 expression and overall survival in multiple cancer types using two databases Survival analysis indicated a significant prognostic role of STAD and LICH. (A and B) OS of PRKD1 expression in STAD patients according to (A) Kaplan-Meier and (B) GEPIA. (C and D) OS of PRKD1 expression in LICH patients according to (C) Kaplan-Meier and (D) UALCAN. Red line: high gene expression, blue and black line: low gene expression GEPIA: Gene Expression Profiling Interactive Analysis, LIHC: liver hepatocellular carcinoma, OS: overall survival, PRKD1: protein kinase D1, STAD: stomach adenocarcinoma, UALCAN: University of ALabama at Birmingham CANcer data analysis Portal

Additionally, the consistency of results from at least two databases confirmed the link between low PRKD1 expression and good prognosis in patients with STAD and LIHC (Kaplan-Meier plotter: HR = 1.71 (95% CI: 1.24-2.37), p = 0.001, Figure 8A; GEPIA: p = 0.01, Figure 8B; and Kaplan-Meier plotter: HR = 1.8 (95% CI: 1.26-2.57), p = 0.0011, Figure 8C; UALCAN: p = 0.0063, Figure 8D, respectively). In conclusion, elevated PRKD1 expression serves as a positive prognostic indicator in KIRC patients, while reduced PRKD1 expression is associated with a favorable prognosis in patients with THCA, STAD, and LIHC.

Evaluation of the methylation status of the PRKD1 DNA promoter

The methylation of the PRKD1 gene promoter is an epigenetic regulatory mechanism that significantly influences the expression of protein kinase D1 (PKD1), a serine/threonine kinase encoded by the PRKD1 gene, and we used the UALCAN database to analyze the methylation status of DNA promoters in BLCA and READ.

**Figure 9:**
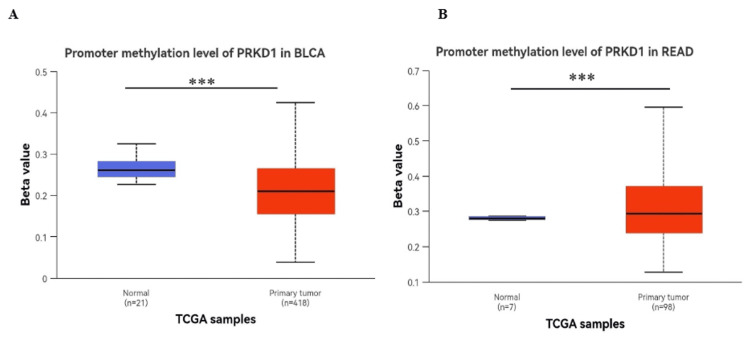
Analysis of PRKD1 gene methylation patterns in normal versus tumor tissues using the UALCAN database The methylation levels of PRKD1 in (A) BLCA and (B) READ. The methylation levels of PRKD1 were significantly hypermethylated in READ (p < 0.001). *p < 0.05, **p < 0.01, ****p < 0.0001 Red: tumor samples, blue: normal samples BLCA: bladder urothelial carcinoma, PRKD1: protein kinase D1, READ: rectum adenocarcinoma, UALCAN: University of ALabama at Birmingham CANcer data analysis Portal

A notable reduction in PRKD1 promoter methylation was observed in BLCA (p < 0.001, Figure 9A), which is inconsistent with its gene expression. Conversely, a significant increase in PRKD1 promoter methylation was detected in READ (p < 0.001, Figure 9C), which is consistent with its expression.

Genetic modifications in PRKD1: a detailed analysis

**Figure 10:**
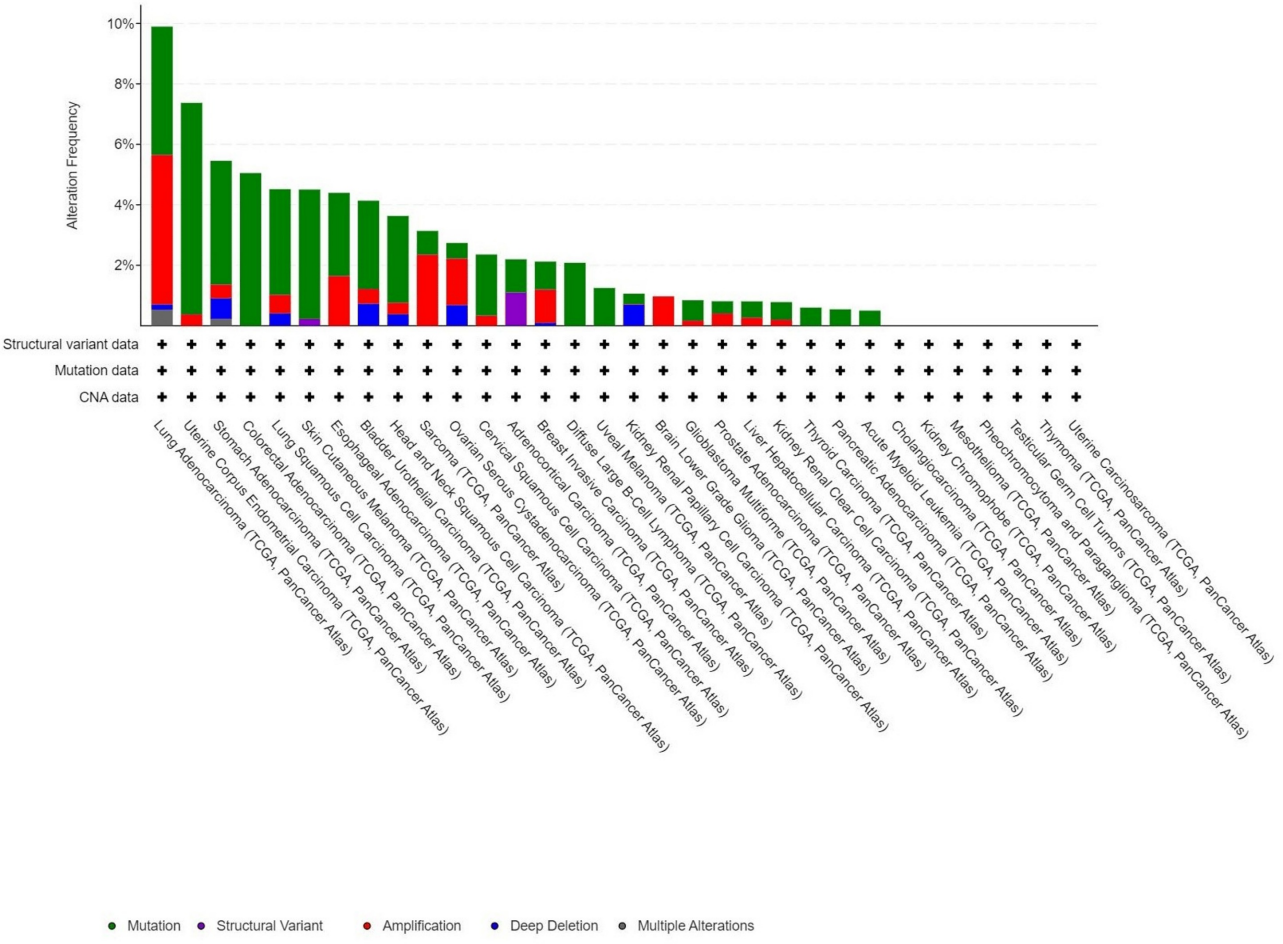
Frequency of PRKD1 genetic alterations in various cancers using the cBioPortal database PRKD1 gene alternation across various types of pan-cancer cBioPortal: cBio Cancer Genomics Portal, PRKD1: protein kinase D1

We investigated PRKD1 genetic variations across multiple cancer types using the cBioPortal platform. Based on TCGA dataset, PRKD1 mutations were detected in 3% of tested samples (10,967 samples from 10,953 patients across 32 studies). The highest prevalence of PRKD1 mutations was observed in endometrial cancer, with 4.24% (24 patients) showing mutations and 0.18% (one patient) exhibiting deep deletions. Additionally, in bladder cancer (BLCA), 2.92% (14 patients) had mutations, 0.49% (two patients) showed amplifications, and 0.73% (three patients) had deep deletions. PRKD1 expression remained unaltered in kidney chromophobe (KICH) and rectal adenocarcinoma (READ) (Figure 10).

**Figure 11:**
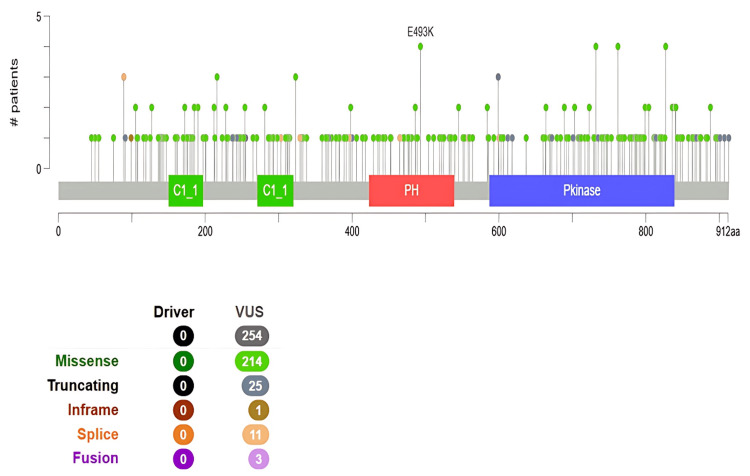
Analysis of PRKD1 mutation types and sites in patients with different cancers using the cBioPortal database This figure displays the types, locations, and numbers of PRKD1 genetic mutations: 912 amino acid residues, two cysteine-rich domains (CysI and CysII), PH, and KD. Green circles: missense mutations, dark gray circles: truncating mutations, brown circles: inframe mutations, dark orange circles: splice mutations, purple: fusion cBioPortal: cBio Cancer Genomics Portal, KD: kinase domain, PH: pleckstrin homology, PRKD1: protein kinase D1

Most PRKD1 mutations are missense, with deep deletions being less common. We observed that the missense variant at position E493K in the PH: PH domain (423-539) was the most frequent alteration among 10,953 samples (Figure 11).

**Figure 12:**
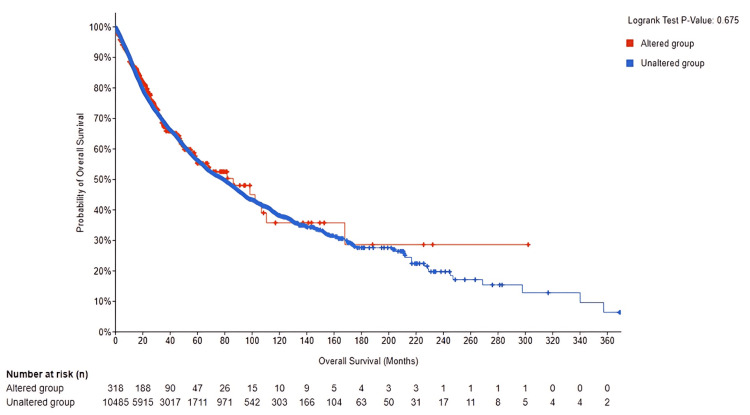
Comparison of overall survival outcomes between PRKD1-altered and unaltered groups using the cBioPortal database At 302 months, 28.62% in the altered group were still disease-free compared with only 6.42% in the unaltered group at 369 months. The altered group showed a non-significant result (log-rank, p = 0.694). cBioPortal: cBio Cancer Genomics Portal, PRKD1: protein kinase D1

To gain deeper insight into the role of PRKD1 genetic changes in cancer development, we examined the overall outcome differences between the altered and unaltered PRKD1 groups. The total number of unaltered PRKD1 group was 318, and the number of events was 100. The total number of altered PRKD1 group was 10,485, and the number of events was 3,413, thereby finding no statistically significant results (log-rank, p = 0.694) of survival between the altered and unaltered group (95% CI) (Figure 12). Genetic alterations examination revealed that only approximately 2% of TCGA samples exhibit mutations.

PRKD1 gene-gene interaction networks

We used UACLAN to inspect genes that are positively correlated (strong or moderate correlation) with our gene in each of the three cancer types to perform gene-gene interactions, protein-protein interactions, and enrichment analysis.

**Figure 13:**
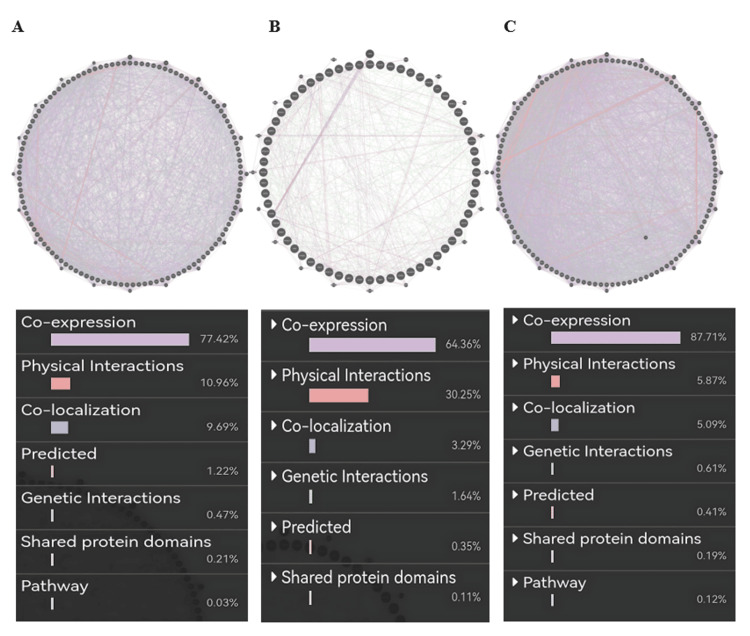
PRKD1 gene-gene interaction networks constructed by GeneMANIA Each node in the figure represents a gene, and the size of the node indicates the degree of interaction. Connecting lines between nodes represent gene-gene interactions, and their colors represent the type of interaction. (A) BLCA. (B) KICH. (C) READ. BLCA: bladder urothelial carcinoma, KICH: kidney chromophobe, PRKD1: protein kinase D1, READ: rectum adenocarcinoma

Gene-gene interaction of PRKD1 in bladder urothelial carcinoma (BLCA) (cutoff: 0.46) showed co-expression of 77.42%, physical interaction of 10.96%, co-localization of 9.69%, predicted of 1.22%, genetic alteration of 0.47%, shared protein domains of 0.21 %, and pathway of 0.03% (Figure 13A). Similarly, the PRKD1 gene in kidney chromophobe (KICH) (cutoff: 0.67) showed co-expression of 64.36%, physical interaction of 30.25%, co-localization of 3.29%, genetic alteration of 1.64%, predicted of 0.35%, and shared protein domains of 0.11% (Figure 13B). Finally, the PRKD1 gene in rectum adenocarcinoma (READ) (cutoff: 0.78) showed co-expression of 87.71%, physical interaction of 5.87%, co-localization of 5.09%, genetic alteration of 0.61%, predicted of 0.41%, shared protein domains of 0.19%, and pathway 0.12% (Figure 13C) using GeneMANIA.

PRKD1 protein-protein interaction networks

**Figure 14:**
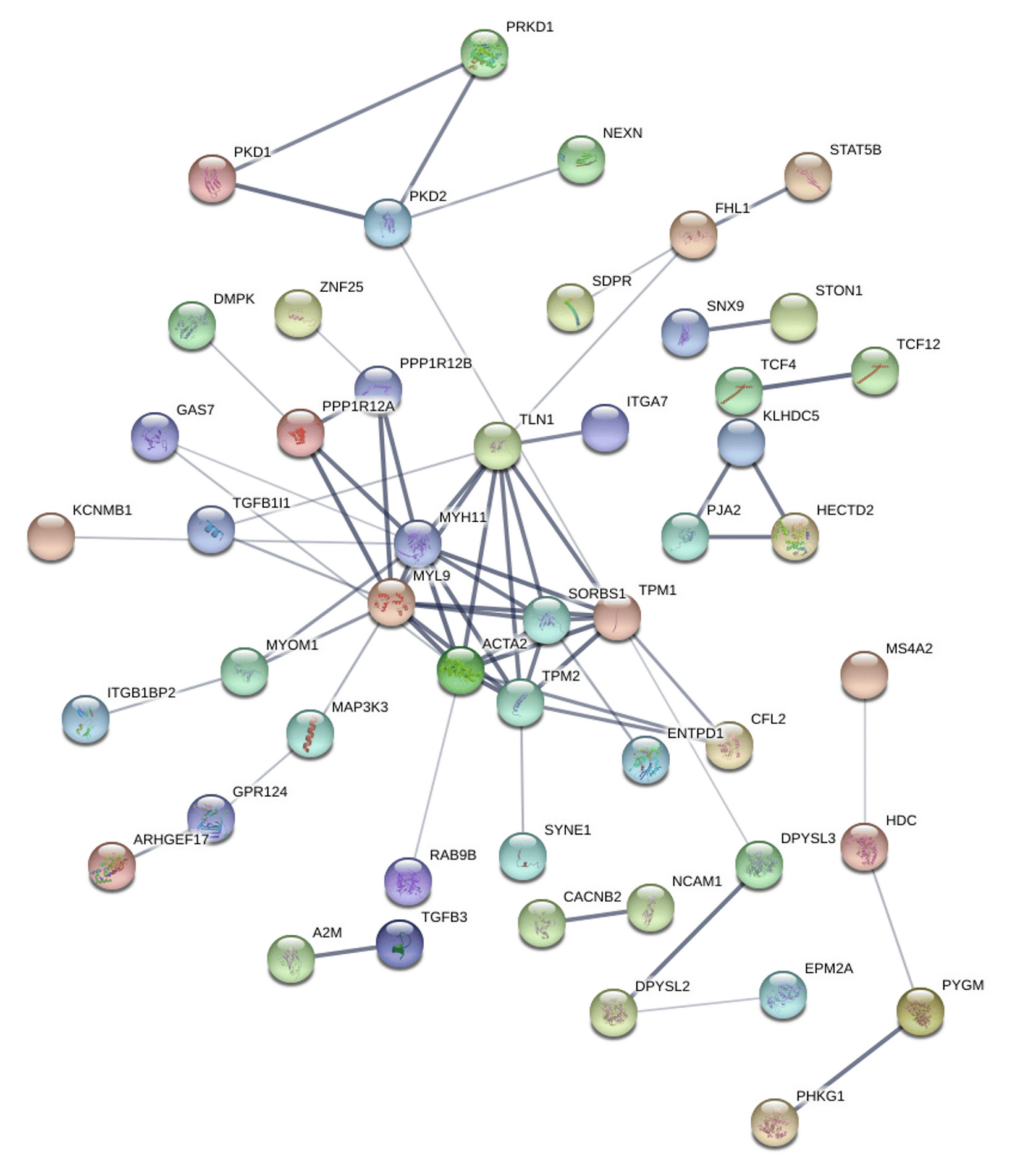
PRKD1 protein-protein interaction networks of BLCA constructed by STRING BLCA: bladder urothelial carcinoma, PRKD1: protein kinase D1

**Figure 15:**
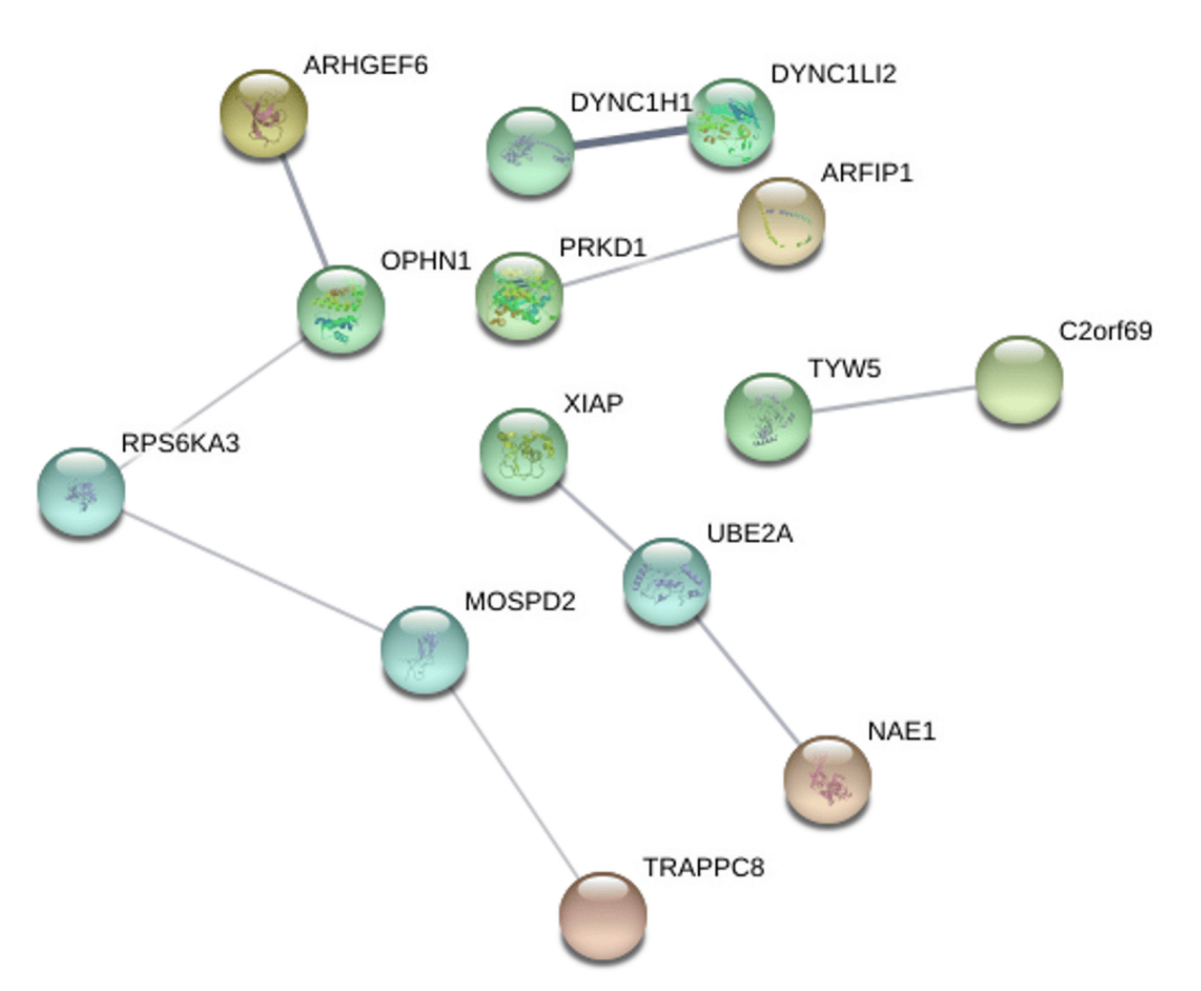
PRKD1 protein-protein interaction networks of KICH constructed by STRING KICH: kidney chromophobe, PRKD1: protein kinase D1

**Figure 16:**
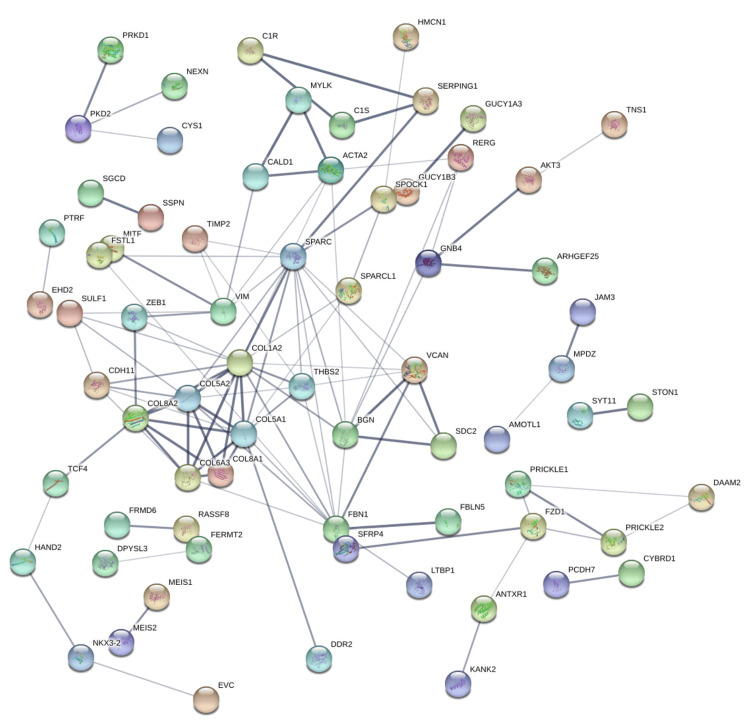
PRKD1 protein-protein interaction networks of READ constructed by STRING PRKD1: protein kinase D1, READ: rectum adenocarcinoma

Protein-protein interaction networks were generated using the STRING database based on genes positively correlated with PRKD1 in KICH, BLCA, and READ (interaction score: 0.4). In the bladder urothelial carcinoma (BLCA) protein-protein interaction network, the number of nodes was 118, and the number of edges was 108 (cutoff: 0.46), and PRKD1 gene had direct interaction with PKD1 and PKD2 (Figure 14). Similarly, the number of nodes in the kidney chromophobe (KICH) protein-protein interaction network was 64, and the number of edges was 9 (cutoff: 0.67), and genes interacting with PRKD1 were ARFIP1 (Figure 15). Finally, in the rectum adenocarcinoma (READ) protein-protein interaction network, the number of nodes were 118, the number of edges was 109 (cutoff: 0.78), and the gene interacting with PRKD1 was PKD2 (Figure 16).

Enrichment analysis of PRKD1 and correlated genes

**Figure 17:**
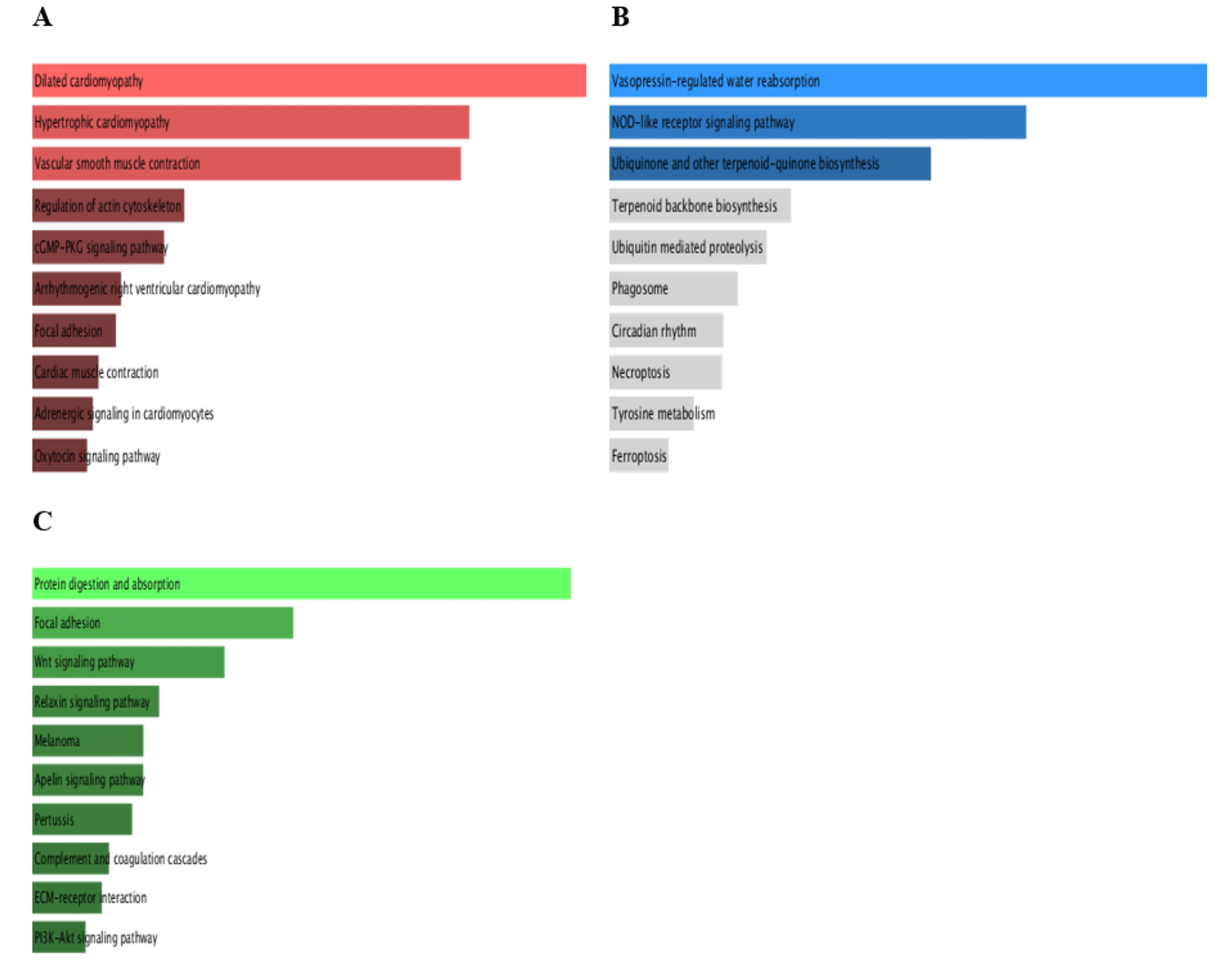
GO term and KEGG pathway analysis using Enrichr This bar plot of the KEGG pathway enrichment analysis of the PRKD1 gene in BLCA, KICH, and READ cancers. (A) Top KEGG pathways of PRKD1 in BLCA. (B)Top KEGG pathways of PRKD1 in KICH. (C) Top KEGG pathways of PRKD1 in READ. BLCA: bladder urothelial carcinoma, GO: Gene Ontology, KICH: kidney chromophobe, PRKD1: protein kinase D1, READ: rectum adenocarcinoma Enrichr: interactive and collaborative HTML5 gene list enrichment analysis tool

Pathway enrichment analysis (PEA) of PRKD1 and the positively correlated genes was conducted in each of the three cancers utilizing Enrichr. We inspected Gene Ontology (GO) and KEGG pathways (Figure 17). In bladder urothelial carcinoma (BLCA), the main significantly enriched pathways were the Rap1 signaling pathway (p > 0.3) and aldosterone synthesis and secretion (p > 0.4). Similarly, the main significantly enriched pathways in kidney chromophobe (KICH) are aldosterone synthesis and secretion (p > 0.2) and the Rap1 signaling pathway (p > 0.5). Finally, the main significantly enriched pathway in rectum adenocarcinoma (READ) was the Rap1 signaling pathway (p > 0.1).

**Figure 18:**
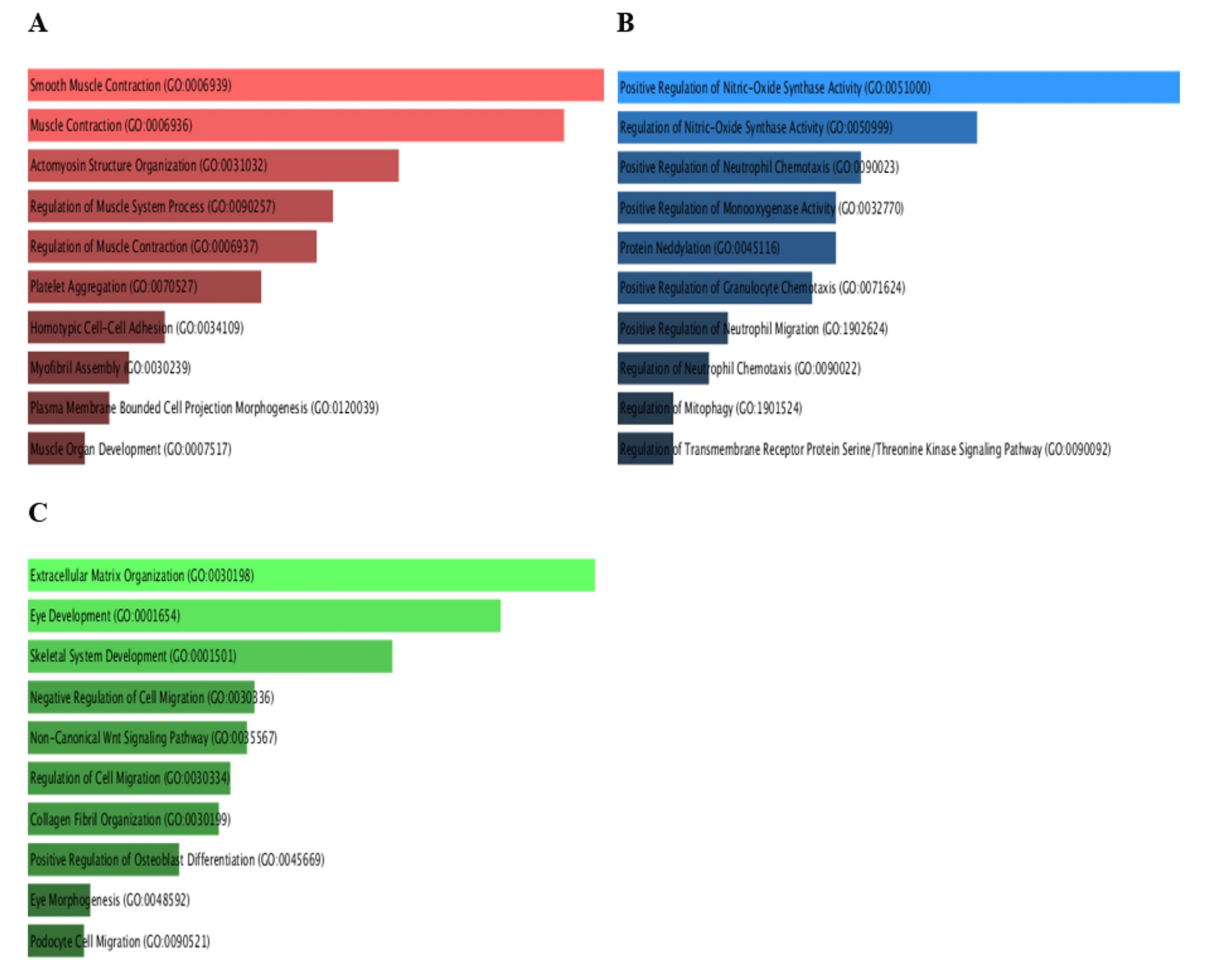
PRKD1 gene ontology and biological process analyses using Enrichr This bar charts of the GO enrichment analysis of PRKD1 genes within biological categories in BLCA, KICH, and READ cancers. (A) The top enriched biological process of PRKD1 in BLCA. (B) The top enriched biological process of PRKD1 in KICH. (C) The top enriched biological process of PRKD1 in READ. BLCA: bladder urothelial carcinoma, GO: Gene Ontology, KICH: kidney chromophobe, PRKD1: protein kinase D1, READ: rectum adenocarcinoma Enrichr: interactive and collaborative HTML5 gene list enrichment analysis tool

GO biological processes of PRKD1 and correlated genes in bladder urothelial carcinoma (BLCA), using cutoff (0.46), showed significant biological processes such as regulation of integrin-mediated signaling pathway (p > 0.004), organelle organization (p > 0.01), positive regulation of cell differentiation (p > 0.03), response to hydroperoxide (p > 0.04), Golgi organization (p > 0.04), and regulation of endocytosis (p > 0.05) (Figure 18A).

Similarly, PRKD1 and correlated genes in kidney chromophobe (KICH), using cutoff (0.67), showed significant biological processes such as regulation of I-kappaB kinase/NF-kappaB signaling (p > 0.005), cellular response to vascular endothelial growth factor stimulus (p > 0.006), peptidyl-serine phosphorylation (p > 0.01), peptidyl-serine modification (p > 0.01), response to hydroperoxide (p > 0.02), peptidyl-threonine phosphorylation (p > 0.02), positive regulation of DNA-binding transcription factor activity (p > 0.02), peptidyl-threonine modification (p > 0.02), positive regulation of blood vessel endothelial cell migration (p > 0.03), and positive regulation of autophagy (p > 0.04) (Figure 18B).

Finally, PRKD1 and correlated genes in rectum adenocarcinoma (READ), using cutoff (0.78), had significant biological processes such as regulation of endothelial cell proliferation (p > 0.001, p > 0.004), regulation of endocytosis (p > 0.006), positive regulation of cell differentiation (p > 0.008), regulation of phosphatidylinositol 3-kinase/protein kinase B signal transduction (p > 0.01), regulation of angiogenesis (p > 0.03), and regulation of blood vessel endothelial cell migration (p > 0.04) (Figure 18C).

Validation of PRKD1 expression using publicly available datasets

**Figure 19:**
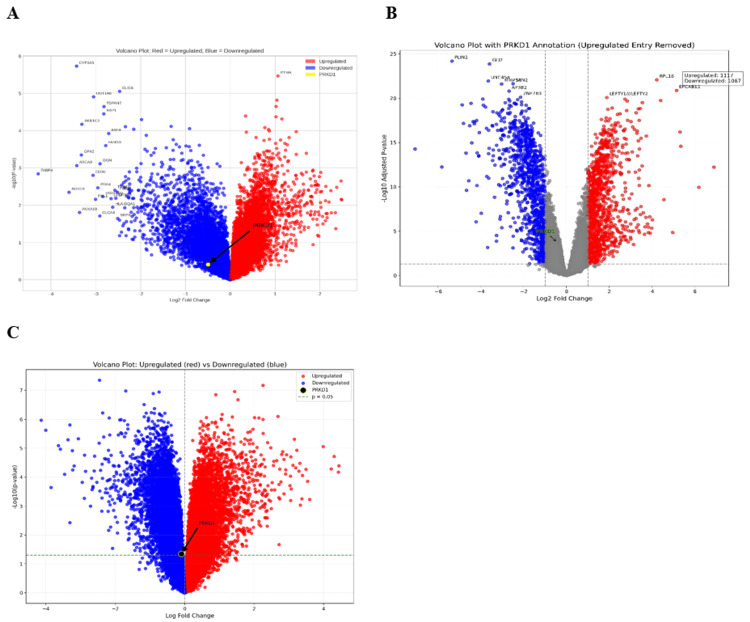
Volcano plots displaying PRKD1 expression in three cancer types (A) BLCA. (B) KICH. (C) READ. The red side of the plot represents the upregulated genes. The blue side of the plot represents the downregulated genes. BLCA: bladder urothelial carcinoma, KICH: kidney chromophobe, PRKD1: protein kinase D1, READ: rectum adenocarcinoma

We analyzed PRKD1 expression across bladder cancer (BLCA), kidney chromophobe (KICH), and rectal adenocarcinoma (READ) using data from the Gene Expression Omnibus (GEO) to validate our previous results. The GEO2R tool was utilized to evaluate PRKD1 expression in these three cancer types, applying criteria of |Log2FC| > 1 and adjusted p < 0.05. Additionally, we generated volcano plots for differentially expressed genes using the SR plot tool. For BLCA, the GSE24152 dataset, which included seven normal kidney tissue samples and 10 urothelial cell carcinoma samples, identified 17,590 differentially expressed genes (DEGs), with 7,560 downregulated and 10,030 upregulated; PRKD1 was downregulated (logFC = -0.49774, adjusted p = 0.674) (Figure 19A). For KICH, the GSE15641 dataset, comprising 23 normal kidney tissue samples and six chromophobe renal cell carcinoma samples, revealed 22,276 DEGs, with 15,223 downregulated and 7,053 upregulated; PRKD1 was downregulated (logFC = -0.4495177, adjusted p = 1.76e-04) (Figure 19B). For READ, the GSE110224 dataset, including 17 colorectal cancer tumor samples and 17 normal samples, identified 54,676 DEGs, with 28,669 downregulated and 26,007 upregulated; PRKD1 was downregulated (logFC = -0.01559186, adjusted p = 0.978) (Figure 19C).

## Discussion

In recent years, the exploration of biomarkers in cancer research has garnered significant attention due to their potential to enhance diagnostic accuracy and prognostic assessments, enabling the early detection of cancer, prediction of response to therapy, and optimization of decisions in clinical practice, leading to a significant reduction in cancer mortality and saving lives [16]. PRKD1 is a serine/threonine kinase belonging to a family of calcium calmodulin kinases (PRKD) alongside two isomers (PRKD2 and PRKD3), playing a pivotal role in key regulatory mechanisms and physiological roles, including cell proliferation, survival, migration, angiogenesis, regulation of gene expression, and protein/membrane trafficking [17], which are intricately involved in pathological processes such as cardiac hypertrophy and cancer progression [18]. The structural and functional similarities shared by the PRKD family, including PRKD2 and PRKD3, underscore the complexity of their individual roles across different malignancies. However, there exists a gap in the literature regarding the comprehensive analyses of PRKD1 and its utility as a cancer biomarker in various cancer types.

This study highlights protein kinase D1 (PRKD1) as a potential diagnostic and prognostic biomarker across multiple cancers using pan-cancer analysis, which has been applied in previous studies [19,20]. Our analysis revealed significant variations in PRKD1 expression, immune associations, genetic alterations, and functional pathways, underscoring its complex role in tumorigenesis.

Using the TCGA dataset, we examined the PRKD1 gene expression profile, which was significantly downregulated in most cancer types, including BLCA, READ, and KICH, compared to normal samples using TIMER, GEPIA, and UACLAN databases. This downregulation unfolds the potential use of PRKD1 as a diagnostic biomarker in these cancers. Previous studies uncovered the downregulation of PRKD1 expression in cancers such as invasive breast cancer [21] and advanced prostate cancer [22]. However, few studies addressed the direct diagnostic role of PRKD1 in cancer.

We used UACLAN to study the correlation between PRKD1 expression and clinicopathological factors such as age, gender, race, and cancer stage for BLCA, KICH, and READ. Notably, Caucasian patients exhibited higher PRKD1 expression than African American patients in both BLCA and READ, suggesting possible racial disparities in tumor biology. Age-specific analysis highlighted that PRKD1 downregulation was more pronounced in middle-aged and older adults, particularly in BLCA and READ, whereas younger individuals showed no significant difference from normal populations. The stage-specific analysis further confirmed the consistent suppression of PRKD1 across all stages in the three cancers, with BLCA and READ exhibiting significant differences across stages, unlike KICH. Significant differences in clinical parameters were highlighted in previous studies, in KICH, BLCA, and READ [23]. These results offer valuable clinical insights that may enhance diagnostic accuracy and treatment decisions and ultimately improve patient outcomes.

To further understand the mechanism underlying PRKD1 downregulation, we analyzed its methylation profile in these cancers. Our findings indicate that hypermethylation of PRKD1 promoter regions in READ is significantly correlated with decreased expression levels, suggesting an epigenetic mechanism regulating its tumor suppressor function. This aligns with previous studies demonstrating promoter hypermethylation as a gene silencing mechanism in many cancer-related genes [24]. In contrast, BLCA was hypomethylated in the promoter region, suggesting that PRKD1 suppression may not be driven by DNA methylation but rather by alternative regulatory mechanisms.

Moreover, survival analysis of PRKD1, detected by at least two databases, manifested its potential role as a prognostic biomarker. Analysis showed that low PRKD1 expression was significantly associated with good prognosis compared to high PRKD1 expression in THCA, STAD, and LIHC patients. In contrast, we found that high expression of PRKD1 significantly correlated with good prognosis compared to low expression of PRKD1 in KIRC patients.

We further investigated the correlations between PRKD1 gene expression and immunological infiltration, particularly CD4+ T cells, CD8+ T cells, macrophages, B cells, neutrophils, and dendritic cells. THCA is often downregulated in active immune responses and is negatively correlated with CD8+ cells. This suggests that lower expression of genes is associated with higher CD8+ levels or activity, potentially enhancing cytotoxic activity of CD8, which promotes an effective immune response. However, it is positively correlated with CD4+ cells, macrophages, B cells, neutrophils, and dendritic cells. Furthermore, STAD and LICH are often downregulated in active immune response and are positively correlated with CD8+ cells, CD4, B cells, macrophages, neutrophils, and dendritic cells.

Finally, KIRC is often upregulated in active immune response and is positively correlated with CD8+ cells, CD4, B cells, macrophages, neutrophils, and dendritic cells. It suggests that the increase in PRKD1 expression enhances the activity of the immune cell and promotes an effective immune response. The immune system controls malignancy, and its suppression fosters cancer progression [25], with immune cells driving smoldering inflammation in the tumor microenvironment [26]. The result suggests that the PRKD1 gene could be a potential biomarker for immune response or a target for therapeutic intervention aimed at enhancing immune response.

Subsequently, we used the cBioPortal platform to analyze genetic variation in the PRKD1 gene in various forms of cancer. Amplification was identified as the most predominant alteration, including BLCA and READ cancers, while mutation was predominant in brain low-grade glioma and sarcoma. Additionally, validation on PRKD1 expression was carried out using the GEO dataset, and results showed that PRKD1 expression was downregulated in BLCA, READ, and KICH.

To further understand the biological significance of these interactions, we performed functional enrichment analysis using Enrichr to identify enriched Gene Ontology (GO) terms and KEGG pathways. The enriched pathways were the Rap1 signaling pathway and aldosterone synthesis and secretion in BLCA and READ, and aldosterone synthesis and secretion in KICH. Recent studies have highlighted Rap1 signaling pathway involvement in cell adhesion, migration, proliferation, and implication in cell invasion and metastasis in different cancers [27]. Studies have also shown the role of the renin-angiotensin-aldosterone system (RAAS) in cell growth, migration, death, and metastasis [28].

The shared biological processes influenced by PRKD1, including endocytosis, endothelial cell migration, and cell differentiation, suggest its significant contribution to maintaining cellular homeostasis and controlling tumor progression. Endocytosis has a crucial role in regulating signaling pathways, receptor degradation, intracellular signal propagation, cell cycle control, apoptosis, and cell fate determination. Subsequently, dysregulation can lead to sustained oncogenic signaling, altered receptor recycling, and enhanced cell motility, enabling uncontrolled growth, survival, and invasion [29]. Additionally, cell migration is essential for proper immune response and tissue homeostasis [30]. Lastly, the association with endothelial migration and angiogenesis further underscores its potential role in tumor microenvironment remodeling, with its suppression potentially promoting abnormal vascularization and metastatic potential. Collectively, these findings highlight PRKD1 as a crucial regulator of cancer-related pathways, emphasizing its potential utility for early detection and targeted therapeutic strategies.

One major limitation of this study is that it relies solely on bioinformatics and computational analyses, necessitating wet laboratory experiments to confirm the diagnostic and prognostic potential of PRKD1. Functional assays, such as knockdown/overexpression studies and tumorigenic assays, are required to validate its role in cancer progression. Another limitation is that it primarily uses TCGA and GEO datasets, which, although comprehensive, may have sample biases or limitations in patient diversity, impacting the generalizability of findings.

## Conclusions

In conclusion, PRKD1 is a promising biomarker for prognosis and diagnosis in many cancer types. PRKD1 expression differed significantly between normal and malignant tissues in BLCA, KICH, and READ according to our pan-cancer research. These results imply that PRKD1 may be used as a diagnostic biomarker, and its levels may help in the early identification and detection of these malignancies. In addition, we identified PRKD1 as a prognostic biomarker in STAD, THCA, and LICH, in which low PRKD1 expression is associated with good prognosis, and in KIRC, in which high PRKD1 expression is associated with good prognosis. Although these findings underscore the clinical relevance of PRKD1, further experimental validation is necessary to establish its future clinical utility.

## Figures and Tables

**Figure 20:**
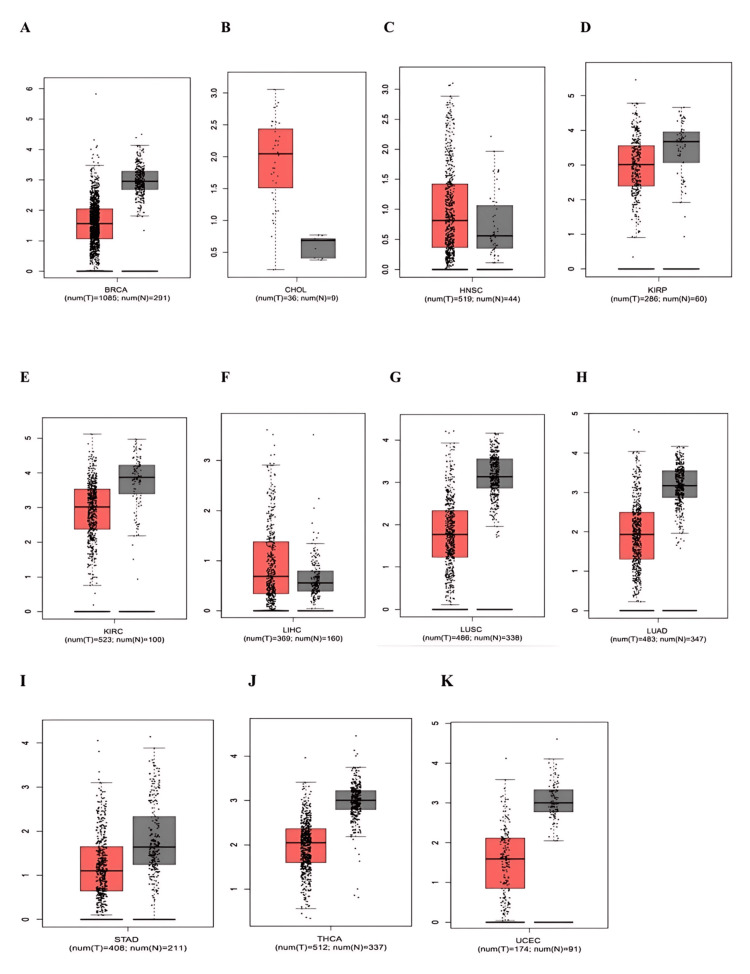
PRKD1 expression in various non-significant cancer types using the GEPIA database (A) BRCA. (B) CHOL. (C) HNSC. (D) KIRP. (E) KIRC. (F) LIHC. (G) LUSC. (H) LUAD. (I) STAD. (J) THCA. (K) UCEC. Red: tumor samples, gray: normal samples BRCA: breast invasive carcinoma, CHOL: cholangiocarcinoma, GEPIA: Gene Expression Profiling Interactive Analysis, HNSC: head and neck squamous cell carcinoma, KIRP: kidney renal papillary cell carcinoma, KIRC: kidney renal clear cell carcinoma, LIHC: liver hepatocellular carcinoma, LUSC: lung squamous cell carcinoma, LUAD: lung adenocarcinoma, PRKD1: protein kinase D1, STAD: stomach adenocarcinoma, THCA: thyroid carcinoma, UCEC: uterine corpus endometrial carcinoma

**Figure 21:**
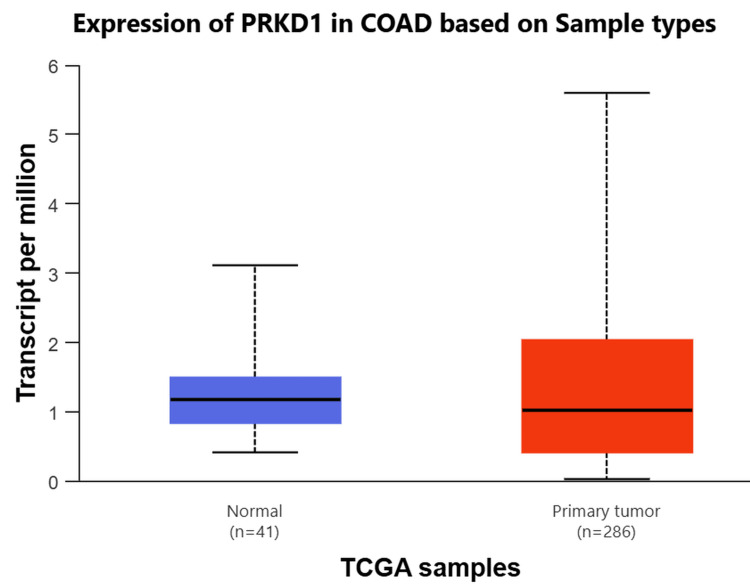
PRKD1 expression in COAD patients using the UALCAN database COAD: colon adenocarcinoma, PRKD1: protein kinase D1, TCGA: The Cancer Genome Atlas, UALCAN: University of ALabama at Birmingham CANcer data analysis Portal

## Appendices

Figure 20 shows PRKD1 expression in BRCA, CHOL, HNSC, KIRC, KIRP, LIHC, LUSC, LUAD, THCA, and UCEC.

Figure 21 shows PRKD1 expression in COAD.
